# Online drug user-led harm reduction in Hungary: a review of “Daath”

**DOI:** 10.1186/1477-7517-10-18

**Published:** 2013-10-02

**Authors:** Levente Móró, József Rácz

**Affiliations:** 1 Department of Behavioural Sciences and Philosophy, Centre for Cognitive Neuroscience, University of Turku, Assistentinkatu 7, Publicum building, FI-20014 Turku, Finland; 2 Department of Addiction Medicine, Faculty of Health Sciences, Semmelweis University, P.O. Box 229, HU-1444 Budapest, Hungary; 3 Institute of Psychology, Eotvos University, Izabella 46, Budapest 1064, Hungary

**Keywords:** Community networks, Drug users, Harm reduction, Hungary, Self-help groups

## Abstract

Harm reduction has been increasingly finding its way into public drug policies and healthcare practices worldwide, with successful intervention measures justifiably focussing on the highest-risk groups, such as injecting drug users. However, there are also other types of drug users in need for harm reduction, even though they pose less, low, or no public health risk. Occasionally, drug users may autonomously organise themselves into groups to provide advocacy, harm reduction, and peer-help services, sometimes online. The http://www.daath.hu website has been operated since 2001 by the “Hungarian Psychedelic Community”, an unorganised drug user group with a special interest in hallucinogenic and related substances. As of today, the website serves about 1200 visitors daily, and the online community comprises of more than 8000 registered members. The Daath community is driven by a strong commitment to the policy of harm reduction in the form of various peer-help activities that aim to expand harm reduction without promoting drug use. Our review comprehensively summarises Daath’s user-led harm reduction services and activities from the last ten years, firstly outlining the history and growth phases of Daath, along with its self-set guidelines and policies. Online services (such as a discussion board, and an Ecstasy pill database) and offline activities (such as Ecstasy pill field testing, and a documentary film about psychedelics) are described. In order to extend its harm reduction services and activities in the future, Daath has several social, commercial, and legislative challenges to face. Starting with a need to realign its focus, outlooks for the upcoming operation of Daath are pondered. Future trends in harm reduction, such as separating harm-decreasing from benefit-increasing, are also discussed. We aim to share these innovative harm reduction measures and good practices in order to be critically assessed, and – if found useful – adapted and applied elsewhere.

## Review

### Introduction

#### Background

In the last decade, harm reduction has slowly but steadily found its way into public drug policies and healthcare practices on national and international levels. Indeed, there is sufficient evidence in support of a variety of harm reduction interventions [1], many of which have been justifiably focussed on injecting and addicted drug users. Successful interventions have yielded clear health benefits, hence helping also the acceptance of harm reduction as an ideology. Lately, harm reduction responses have been expanded also into the broader perspective of caring for the health, social, and economical needs of problem drug users, especially those who are socially excluded [2]. However, there are also other, notably large drug user groups in need for harm reduction, who should not be ignored or left aside, just because they pose less, low, or no public health risk. Non-injecting and non-addicted drug users may need harm-reducing measures for different types of drugs, but for partly similar drug-related problems concerning purity, methods of administration, and psychosocial issues. In many cases here, harm reduction can take an immaterial form, such as the dissemination of factual and experiential information.

As the amount of drug-related information exchange is skyrocketing on the Internet, emerging novel technologies (e.g., image databases, video sharing, and social networks) may create new opportunities for online harm reduction. Compared with the actual “one-on-one” physical harm reduction work out there in the “real world”, drug-related online information may typically reach relatively large numbers of people by relatively small resource investments. Characteristic trends in the emerging information society have been indeed utilised by governmental organisations (GOs), nongovernmental organisations (NGOs), as well as unorganised interest groups, such as peer-helping drug user communities [3,4]—but not at all without criticism. Especially in those cases when drug users share information between each other, information that may decrease drug-related harm (e.g., warnings about the circulation of bad quality drugs) can easily mix with information that may facilitate drug use (e.g., hints about the sellers of good quality drugs). It can be reasonably claimed that such drug information may increase drug use prevalence and thus increase also *potential* drug-related harm. However, carefully selected and communicated harm reduction information – while it may inevitably increase drug use prevalence to some extent – should be able to decrease *actual* drug-related harm. It is also conceptually disputable whether a lower prevalence rate should be a goal in itself—or is it actually a lower harm-to-prevalence ratio that should be actually pursued?

Due to the hiding nature of illicit drug use, it is not always possible to receive feedback, or to assess the outcomes and effectiveness of certain harm-reducing interventions. Hence, a feasible and pragmatic way of advancing harm reduction is to merely share “best practices” among interested parties. Admittedly, many of these practices are unproven and yet to be assessed, but nevertheless they could be at least detailed and listed in order to facilitate an evaluation process later on.

#### Drug use, harm reduction, and public attitude in Hungary

By lifetime prevalence of drug use and by problem drug use rate, Hungary resides generally in the low to lower middle range within the European Union [2]. In 2007, the most widely used illicit drugs were cannabis, ecstasy, and amphetamine, with their lifetime prevalence rates of 19%, 5%, and 4% respectively in the young adult population (of age 18–34 years) [5], and 14%, 5%, and 4% respectively in the student population (of age 15–16 years) [6]. However, consumption of legal drugs is even more popular: Combined use of medicines and alcohol (12%), sedatives/tranquilisers without medical indication (9%), inhalants (8%), and nitrous oxide (5%) take the second to fifth places in the lifetime prevalence list among the 15–16 year-old students. Lifetime prevalence rates for psychedelic drugs are quite marginal: 3% for LSD or other hallucinogens, and 1% for magic mushrooms [6].

Harm reduction has been officially incorporated into Hungary’s latest “National Strategy for Tackling the Drugs Problem 2010–2018” [7]; however, this strategy was repealed already in 2010 by a subsequent government, and so far no new strategy has been provided instead. The bundle of treatment, care, and harm reduction was one out of the three pillars in the repealed national drug strategy (the second pillar was prevention and community interventions; the third pillar was supply reduction). Even though the idea of harm reduction as an independent fourth pillar was rejected during the planning phase, and despite its modest appearance in the drug strategy, there is a revived political debate on rewriting the whole drug strategy with an even more reduced emphasis on harm reduction. This forthcoming change would presumably make the operating environment harder for the dozens of harm reduction NGOs, many of which are providing needle and syringe programs and outreach services under the umbrella organisation MADÁSZSZ.

In spite of the country’s modest drug problem situation, public attitude toward drug users is remarkably negative. A 2003 survey that asked the general population about their preferences for certain groups (e.g., HIV positives, homosexuals, alcoholics, mentally ill, or Roma people) found out that drug users were the most discriminated group in the comparison: More than 80% of the survey participants rejected the idea of living next door to a drug user [8]. This negative attitude and moral panic is probably largely shaped and fuelled by the public media that generally does not differentiate between problematic and non-problematic drug use. It also maintains the stereotypical image of drug users as either dangerous criminals or addicted “junkies”. These terms and similar allegories are also reflected in political speeches, strengthening further the reluctance to establish a compassionate and tolerance-based harm-reducing approach in public drug policies.

#### Aim of the study

The current article reviews the profile, structure, and operational practices of “Daath”, an online group of drug users in Hungary. The word “Daath” (Hebrew: דעת; phonetic spelling: dah’-ath; meaning: Knowledge), originating from the Kabbalah, was chosen to denote the importance of knowledge in seeking mystical experiences. Self-described as the “Hungarian Psychedelic Community”, this unorganised group has a special interest in hallucinogenic and related substances, as well as a strong commitment to the policy of harm reduction by its various peer-help activities. As of today, the http://www.daath.hu website serves about 1200 daily visitors, and the broader online community comprises of more than 8000 registered members. Based on a decade of participative observation, a comprehensive summary and retrospective review of Daath’s drug user-led online services and offline activities from the last ten years are presented in the following. By sharing here, innovative measures and tentative harm reduction best-practices are hoped to be assessed and possibly adapted also elsewhere.

### The Daath community

#### Overview of activities

As a drug user activist group – albeit an unorganised one – Daath can be best profiled by its peer-provided services. On the 20-item list of drug user group services compiled by Goossens [9], four items match Daath’s online activities: “awareness and information”, “peer support for drug users”, “psychological support related to drugs consumption”, and “accurate information through regularly updated Internet sites”. Moreover, the limited offline activities of the Daath community belong to three major categories in Goossens’s classification: “support and information for party organisers”, “educational and peer support work in the party scene”, and “drug checking”. Apart from these offline activities, Daath does not provide physical services for drug users, as it has neither organisational nor financial resources for providing face-to-face peer-help. Albeit some active members of the community are involved with advocacy and policy-making processes in the health/drug field, their activities are neither in direct connection with nor specifically representing Daath.

#### History

Although Daath can be seen a spontaneously built-up drug user community, a closer look at its developmental history reveals an increasingly coordinated effort started out by a few individuals. In spite of their diverse ideological beliefs and backgrounds, all these early individual contributors had a strong common interest in psychoactive substances from the mid-1990s. The first version of the website in 2001 compiled together a large amount of drug information available in its native language: The former Hungarian Drug Pages, some writings from Lycaeum.org, translations from Erowid.org, and other online articles on the topic. The original webpages were followed in 2002 by a second version with a distinctive visual style and a discussion board: the Forum.

In its earliest stage, the Daath website was operated by its founders, whose main task was to occasionally add more drug-related content. However, as the new Forum gained popularity, the quality of discussion had to be maintained by new volunteers acting as Forum moderators. Soon Daath formed a lively online community that became a primary resource and virtual meeting point of a specialised subgroup within the drug user subculture. After about one year, a need to meet “in real life” emerged within the community members, and small personal networks started to form within Daath. However, as personal communication (by email, mobile phones, and instant messaging) had increased, public interaction on the discussion board decreased between those who kept contact with each other also outside the Forum. This phenomenon had led to the forming of an “inner circle”, a subgroup that became more connected internally, but invisible to other public discussion board participants [10].

Concerning the profile of Daath, a notable change of direction has started in 2004. Around this time, the composition of Ecstasy pills became unpredictably variable, with some pill batches causing headaches, nausea, vomiting, overstimulation, and other physical harm to the users. This changed situation called for urgent harm-reducing action in order to tackle with this problem. By establishing an Ecstasy pill database and increasingly providing information on new synthetic drugs (NSD), Daath started to focus also on harm reduction for recreational party drug users. On the basis of Forum discussions, individuals in this new subgroup seem to differ from typical psychedelic drug users primarily in their younger age, less interest in psychedelic drugs, and more frequent use of stimulant drugs.

#### Community roles

By their roles taken within the community, Daath members can be classified into four groups: (1) contributors, (2) readers, (3) registered members, and (4) discussing members. In the following, these groups will be described in a bit more detail, along with some descriptive statistics:

##### Contributors

Being an informally operated “unorganisation”, the Daath community has no appointed staff for performing various tasks. However, in each stage of the development, significant amounts of volunteer work had been contributed to enhance Daath with web design, site programming, server maintenance, text translating, information compiling, Forum moderating, and content editing.

##### Readers

As of now, the Daath website receives traffic of around 1200 unique visitors per day, a 20% increase from the previous year. However, this all-time visit record was most probably due to the huge interest in the designer drug mephedrone (4-methyl-methcathinone; 4-MMC). Being an online media without borders, Daath is read and commented also by Hungarian-speaking minorities in neighbouring countries, as well as expatriate Hungarians worldwide. This explains top foreign visits, which come from Romania (1%) and the Slovak Republic (1%).

##### Registered members

Summed with last year’s 1187 new registrations – a 23% increase from the previous year – a total of 8477 persons registered on the http://www.daath.hu website since February 2002. On the Daath discussion board (the Forum) these members are identified by self-chosen nicknames (pseudonyms).

##### Discussing members

It is to be noted that certainly not all registered members participate in the discussions. The number of active participants is much lower: 59% of registered members never made any comments on the Forum, 12% commented only once, and 5% only twice. For example, last year’s active discussion – comprising of 10205 comments – had been contributed by a total of 564 individual Forum members. Although no demographic surveys were ever carried out in the community, Forum participants appear to be most typically young men in their early and mid-20s. On the basis of self-references, the vast majority of these Forum participants are non-problematic, occasional users of mostly psychedelic and semi-psychedelic substances.

#### Psychedelic, semi-psychedelic and non-psychedelic drugs

Daath focuses particularly on hallucinogenic and related substances that are mainly used for a “psychedelic” purpose. Coined in 1956 by psychiatrist Humphrey Osmond, the term refers literally to “mind manifestation” (or popularly to “mind expansion”): A characteristically altered state of consciousness with a subjective experience of increased perception and apperception, dissolving of personal boundaries, and feelings of unity [11]. Substances that may provide these types of experiences include primarily the four major hallucinogens: LSD (lysergic acid diethylamide), DMT (N,N-dimethyltryptamine from plant species, or in the *Ayahuasca* decoction), mescaline (from *Lophophora* and *Trichocereus* spp. cacti, commonly known as *peyote* and “San Pedro”), and psilocybin (from *Psilocybe* spp. mushrooms, the so-called “magic mushrooms”). In addition, salvinorin-A (from *Salvia divinorum*, “diviner’s sage”) and LSA (lysergic acid amide, from *Ipomoea violacea* “morning glory” and other similar plant seeds) are also often counted as psychedelic drugs [12].

Certain psychoactives are arbitrarily classified as “semi-psychedelic” drugs by Daath. Such substances that in larger doses may produce minor psychedelic-like effects are THC (Δ-9-tetrahydrocannabinol, from *Cannabis indica* spp. “marijuana” hemp) and MDMA (3,4-methylenedioxy-methamphetamine; “Ecstasy”). In addition, certain rare or marginal hallucinogens, such as various phenethylamines (e.g., 2C-B) and tryptamines (e.g., 5-MeO-DMT), dissociative anesthetics (e.g., ketamine and nitrous oxide; N_2_O, “laughing gas”), *Amanita* spp. mushrooms (e.g., “fly agaric”), and ibogaine (from *Tabernanthe iboga* shrub) are reported to produce psychedelic-like effects [13].

Drugs that are not considered psychedelic – such as heroin and opioids, cocaine and amphetamines, prescription drugs, and inhalants – are generally excluded from Daath discussions. One reason for this is to express a clear separation between the preferred “mind expanding” drug subculture and the despised “mind contracting” usage of depressants and stimulants. These latter two drug groups are generally associated with hedonistic, egoistic, pleasure-seeking, or self-harming usage patterns and habits. Although this separation on the basis of a drug group is rather arbitrary and unjustified, it still implies a discouragement toward those drugs that are generally considered as “more dangerous” because of the risks of overdose, health damage, or addiction. But more importantly, the separation also sends a discouraging signal toward negatively considered behavioural patterns, such as excessive or reckless drug use—no matter which drugs are actually involved.

#### Policies

##### Guidelines

Daath enforces its self-developed regulations for guiding behaviour on the Forum. All new discussion board members are required to read through, accept, and comply with these norms. The guidelines serve in order to keep the website focussed on its primary topic (i.e., psychedelics), to ensure high quality discussion, and to avoid illegal or otherwise undesirable content. Upon registration by choosing a unique nickname, a summary of Forum rules are sent for acceptance to the registrant by email. A brief summary of avoidable content, with some items highlighted in red, is clearly shown on the comment-writing webpage as a reminder of proper behaviour.

##### Forum entry test

To ensure high quality discussion for those interested in the topic of psychedelics, the Daath Forum requires passing an online entry test after registration. In order to prove genuine interest and willing to acquire basic knowledge on psychedelic drugs, new registrants must fill in a 12-question test form. For each new registrant, entry test questions are drawn randomly from a pool of 64 available questions, and displayed with three-choice answer options. Questions are asked about the basics of psychedelic drugs, harm reduction, and Forum guidelines. In case of one or more incorrect answer, the test may be retaken immediately with another set of questions. Passing the entry test – i.e., giving all answers correct – will entitle the registrant to add comments on the Forum as a new discussing member. By adjusting the number of questions, the overall pass/fail rate can be kept around a desired 50%. This threshold has been considered adequate to let in those who are interested enough, and to keep out the rest of the registrants.

##### Limitations

In order to keep a self-made standard on discussion readability, some passive technical rules are set to limit Forum interaction. Daath’s custom-made discussion board software imposes quantitative limits on comments, concerning length (4096 typed characters), interval (3–5 minutes), and amount (8–12 comments per day). The goal of this policy is to guide toward moderate discussion by encouraging fewer but better considered comments from Forum participants, thus helping to avoid a “chatting” discussion style. The daily limitation also carries an implicit “send-away” message: Obtaining drug-related information indeed deserves attention and time, but the topic of drugs should not be a central issue in Forum participants’ lives. Hence, after a certain amount of daily activity, Forum users are automatically restrained from participating in the discussion until midnight that day.

##### Moderation and self-moderation

Daath Forum topics are currently monitored by four administrators who ensure that the discussion complies with the guidelines. In general, discussion board moderation is kept as minimal and flexible as needed, but nevertheless consistent with its principles. Beside corrections of the technical kind (e.g., relocating off-topic messages, or removing accidental duplicates), Forum comments may be actively modified or removed if they do not meet a set of explicitly defined content rules. These rules concern primarily the acquisition of *any* psychoactive drugs (regardless of their legal status), offences against the law or another person, overt verbal aggression, obscene language, commerciality, and political issues except drug policy and peace movements. First-time rule breakers are simply reminded of the Forum rules in a neutral manner. Offending comments may be removed in part or in whole, with a possibility to specify a reason for removal in red colour. However, in very rare cases of continuous and deliberate offences, delinquents can be temporarily or even permanently removed from the Forum.

##### Minors (under 18 years old)

Unlike many other drug-related websites, Daath does not restrict minors (under 18 years old persons) from accessing the provided drug information, nor from participating in the discussions. By age 15–16, 13% of adolescents in Hungary have already made their first contact with cannabis, and 7% with other illicit drugs [6]. Therefore, Daath’s policy holds that it would be an act of irresponsibility to prevent or limit anyone’s access to accurate drug information and peer-help, especially of those in the most vulnerable age group. In practice, the community observably shows more care toward its younger members, and often provides preventive or disadvising guidance to them.

##### Commerciality and promotion

Being operated by private persons, Daath is ideologically and financially independent from governmental organisations, political parties, business companies, and other sponsors. Even in spite of repeated business proposals and offers from legally operating headshops, smartshops, and growshops, Daath refuses to exhibit advertisements for any psychoactive substances or related products on its webpages. Daath holds that psychoactive drugs, be they legal or illegal, are not suitable for everyone. Thus, drugs should not be distributed to others without taking responsibility of the novice who is being introduced to this new realm. Any ethically approvable drug commerce should not only include detailed instructions on exact dosage and least harmful routes of administration, but should be also preceded by an evaluation of the buyer’s psychological maturity, preparedness, and responsible attitude toward drug use.

### Harm reduction: services and activities

#### Online services

##### Substance information

Daath’s main emphasis is on the dissemination of reliable, fact-based, and up-to-date drug information that reduces harm. For many years, its Substance Information webpages have been a widely known and referred source in Hungary, compiling data on dosage, usage, and effects of 17 mainly psychedelic substances. In addition, a total of 14 drug information flyers by the harm reduction organization DanceSafe (http://www.dancesafe.org) had been translated into Hungarian and published within the Substance Information section in summer 2007. Volunteers of the Daath community have also taken part in editing drug-related entries of the Hungarian-language Wikipedia (hu.wikipedia.org), which is considered by many as an authoritative source of drug information—regardless of its actually unasserted reliability.

##### Drug library

The Daath Drug Library aims to collect, organise, and publish drug-related documents available in Hungarian. It currently holds 199 writings in ten categories: Tutorials, Short Writings, Studies, Presentations, Legal Texts, Books, Articles, Essays, Interviews, and Experience Reports. These writings are originated both from readers of the website and from outside sources, disseminating a wide range of drug information best practices, drug-related study results, current drug laws and related regulations, as well as works of literature and journalism on the drugs topic. Based on readers’ ratings, the most popular library item is the “Harm Reduction Guide” [14] compiled by Daath’s editor-in-chief. This library item is closely followed in popularity by book excerpts from Hungarian-born Canadian psychotherapist Andrew Feldmár, who openly talks about using LSD as a therapeutic tool in his practice in the 1970s [15].

##### Discussion board (the forum)

The online core of the Daath community is its public discussion board: the Forum. As of October 2011, there are 249 active discussion topics hosted on the Forum in nine categories: Society, Culture, Philosophy, Internet, Writings, Psychedelic Drugs, Other Substances, Practices, and Opinion. Categories are fixed in number, but new topics are being opened by the administrators, albeit rarely. From the beginning of operation in February 2002 until February 2011, a total of 111769 comments were made by a total of 3143 persons on the Forum.

##### Ecstasy pill database

In terms of harm reduction, Daath’s most important online service is definitely its Ecstasy pill database, a collection of pill descriptions (by colour, logo, and shape) along with subjective experience reports concerning that particular pill. As mentioned above, this service was started in response to those situations where pills sold as Ecstasy contained no MDMA at all, but instead amphetamines, anabolic steroids, or mCPP. Since its launch eight years ago, Daath’s Ecstasy pill database has been steadily growing, and it currently holds a total of 2708 brief reports about 889 different pills from 401 individual contributors. In the database, 66% of the pills are categorised as producing MDMA-like effects, 23% as dangerous and non-MDMA-like, and 11% as having no effects at all. In order to avoid criticism for the “promotion” of high quality Ecstasy pills, the database does not allow quantitative scoring of pills on a scale, thus making it impossible to explicitly search for “good” pills.

Despite practical limitations (e.g., similarly looking batches of pills may contain different active substances), the Ecstasy pill database service has been appraised by drug users as utterly helpful in drug-related harm reduction. The pill database also aims to raise awareness about the dangers of consuming pills with unknown origin and composition, thus promoting a cautious attitude and informed risk taking. In this way, harm can be minimised when Ecstasy users do query the database or browse the discussion group either before purchasing a pill or before consuming it. Users have repeatedly reported on the Forum that they have successfully avoided buying fake Ecstasy pills by using the Daath pill database. Upon finding out about a fake pill only after purchasing, some other users reported that they discarded the pill instead of consuming it—or returned their bad Ecstasy pill to the dealer with complaints.

##### Experience reports

Compared to the actual proportions of drug-related harm, negative stories about illicit drugs – including the topics of addiction, violent crimes, mental problems, overdoses, accidents, and death – are blatantly overrepresented in the Hungarian public media [16]. Characters and events – be they real or fictional – are utilised to build up sensationalist “stories with a moral” that often aim to terrify and deter from illicit drug use. It is to be noted that the use of legal drugs, such as alcohol and tobacco, is simultaneously presented in the public media as a lifestyle and as “part of the culture” (or even “part of the national heritage”), despite their deleterious health effects on both national and global levels. From a harm reduction point of view, such media policies could be considered dangerously counterproductive for a number of reasons. Although drug-naïve subjects in their drug-free microenvironments may be possibly deterred from experimenting with drugs, but the message of “Say No To Drugs” is much less convincing for those who have already observed or experienced non-problematic drug use. Moreover, previous beliefs about the effects of a drug may play an important mediating role in the adoption of attitudes and expectations about that particular drug. Contrary to this, Daath wishes to represent typical and realistic drug use situations among a presumably average group of young drug users (as opposed to the media-overrepresentation of *problematic* drug users). Submitted experience reports, be they positive or negative in their outcome, are put to display by Daath without content modification. It may be indeed argued that negative drug experiences are less likely to be reported publicly on Daath (or elsewhere), perhaps because of the shame felt, or of a discontinued interest due to unfavourable happenings. While this may be true, the role of Daath is certainly not to counterbalance, but to remain an open media channel that enables public submission of anonymous drug experience reports.

##### “Earlier warning system”

Changes in drug use trends, as well as new trends are quickly detectable on Daath’s discussion board. In the last few years, Daath members had reported about adverse effects of certain Ecstasy pill batches that were later identified as adulterated with mCPP, about cannabis laced with fine-grained quartz sand causing lung problems, and about the appearance of GBL as a substitution of GHB in night clubs—all before public media reports. In addition, Daath members had also reported about unexpected cannabis-like effects of various “Spice” smoking mixes long before laboratory measurements revealed synthetic cannabinoid agonist compounds in those mixtures. Forum discussion revealed also the usage trend of the then-new party stimulant BZP that had not been previously seized in Hungary. Clearly, first-hand information from drug users is valuable for not only the drug users themselves, but also for institutions concerned with monitoring new trends in drug use. Such a network is the Early Warning System operated by the Reitox national focal points, reporting to the European Monitoring Centre for Drugs and Drug Addiction (EMCDDA). Not even being a NGO, Daath had difficulties in getting approved as an official participating partner in this national network until recently. Nevertheless, Daath has been operating its own “Earlier Warning System” for drug users by issuing special alerts about dangerous substances, as well as by temporarily publishing a monthly newsletter about the very alarming drug market change trends in 2009.

##### Special activities and miscellaneous campaigns

From time to time, Daath organises special activities and campaigns for its community members. In 2006, 12 active Forum members won free membership of the Hungarian Civil Liberties Union (HCLU) in a raffle sponsored by an anonymous patron. Another campaign was the “Cannabis Fasting” competition, where discussion board members documented online their one-month voluntary abstinence from marijuana. In 2008, a community fundraising was organised for the benefit of Erowid (http://www.erowid.org), one of the largest international websites dedicated to psychoactive drugs. With a total of 390 USD raised, Daath became a Fellow-level supporter of Erowid, in appreciation for the website’s mission of “providing access to reliable, non-judgmental information about psychoactive plants, chemicals, and related issues” [17].

#### Offline activities

##### Party service

Party services are groups of volunteers who attend electronic dance music events (such as parties, raves, or festivals) to offer free drinking water, snacks, drug information, and psychological help for the party-goers. These services may be ad hoc assembled, or operated by local NGOs, who may be partially funded by local GOs or corporate sponsors for the costs of transportation and provided goods. Distribution of free drinking water is often considered as an essential service, because some venue owners may turn off cold water taps in the restrooms, in hope of an increased profit on (often overpriced) mineral water sales at the counter. This unfortunate practice could be dangerous for drug-using party people: The combination of metabolism-increasing stimulants, higher ambient temperatures at hot and crowded venues, and prolonged dancing without rest and without drinking enough liquid may lead to overheating of the body. MDMA-related hyperthermia has been a cause of emergency room visits and, albeit occurring rarely, several deaths [18].

Hallucinogenic drug use in a party environment may occasionally turn into a *bad trip,* especially for unprepared and non-experienced persons with an unstable worldview and an irresponsible attitude toward mind-altering substances. Such situations need special handling methods: Calming down the person by friendly talk, disrupting his/her negative thought patterns, showing care and attention, and providing a safe and relaxing environment until the drug effects wear off. Being experienced with hallucinogens, some Daath members occasionally volunteer in providing such *psychedelic emergency* (PsyEm) services at parties and festivals.

##### Ecstasy pill/powder testing

In close connection with its Ecstasy pill database and party services, Daath also promotes the field testing of pills and powders. In 2009 in Hungary, as low as only 1.4% of all seized Ecstasy pills contained MDMA alone, the rest having fully or partially other active or inactive substances in them. The most often detected other substances were amphetamine and mCPP, but there is a notable appearance of substances previously not found in pills, such as methamphetamine, GHB, MDPV, 4-fluoroamphetamine, 2C-B, butylone, and ketamine [19]. For probing Ecstasy pills and other psychoactive substances in the field, many commercial testing kits use the Marquis reagent. A drop of this mixture of sulphuric acid and formaldehyde turns purple-black when comes in contact with a tiny scraped bit of a pill or a small amount of powder that contains MDMA. However, this testing method can be used with certainty only to detect pills or powders that are *not* containing MDMA-like substances (i.e., when no colour change occurs). A purple-black colour reaction may also indicate the presence of other substances (such as dextromethorphan; DXM), and/or it cannot exclude the possibility that the tested material contains other ingredients besides MDMA. It is also to be taken into account that criticism against Ecstasy testing claims that the procedure may give a false sense of security for the users, as if drug use could be made *safe* – not only *safer* – by these methods. While this may hold true, and while the Marquis reagent’s precision is clearly inferior in comparison with laboratory chromatography, this simple field testing method may still remain an inexpensive and easily available “better than nothing” option for party drug users.

##### “Psychonauts” - a documentary film

In 2006, a movie project was carried out by core members of the Daath community. Titled “Psychonauts”, this 41-minute interview documentary film by director András Kovács M. introduces nine persons who had formerly used or currently use psychedelic drugs. In thematically organised blocks separated by aphorisms, these young adults reveal their personal opinions, experiences, and thoughts about psychedelics-related issues. The documentary is freely downloadable in several formats from the Daath website and may be watched also from YouTube [20]. The Hungarian-language video was originally subtitled only in English, however – to the filmmakers’ surprise – international fans of the movie had voluntarily provided Czech, Finnish, German, Russian, and Spanish translations. There is also a DVD version available, dedicated to the inventor of LSD, Albert Hofmann (1906–2008).

#### External relations

##### GOs and NGOs

With the exception of occasionally providing data for the EMCDDA on request by the Reitox Hungarian National Drug Focal Point, Daath has no formal links to governmental organisations involved in drug policy making. Instead, Daath supports the activities of the HCLU, a human rights NGO working also toward a drug policy reform in Hungary. Members of the Daath discussion board also help with HCLU’s Media Monitor Program, which aims to improve the quality of media coverage on drugs by providing fact-based drug information to journalists. Some Daath members are known to participate in other drug-related local NGOs, such as the Blue Point Party Service, the MGTSZ Party Depot, the pro-cannabis Hemp Seed Association, and the research-oriented Multidisciplinary Society for Psychedelic Studies.

##### Studies and surveys

Daath Forum members have been repeatedly asked to participate in quantitative and qualitative drug studies as anonymous respondents or interviewees. To date, these studies include a qualitative study in sociology about drug use habits and family relationship dynamics [21], a master’s thesis in cultural anthropology about the structure, activities, and interpersonal relations within the Daath community [10,22], and another master’s thesis in religious studies about the sacramental use of hallucinogenic substances [23]. The relationship between psychedelic drug use, life quality, and spirituality has been studied by a master’s thesis in health sciences [24], and subsequently published in a peer-reviewed journal [25]. Moreover, by the request of the Reitox Hungarian National Drug Focal Point, a short statistical report on the consumption habits of mephedrone [26] was compiled and included in the 2011 national report to the EMCDDA [27]. At the moment, there is an ongoing survey that gathers data on *Salvia divinorum*’s general and bodily effects.

##### Public media

Contrary to its low public profile, Daath has been mentioned in virtually all formats of the Hungarian public media: daily political newspapers, weekly magazines, state-owned and commercial TV channels, and even in a speech at the Parliament. Especially in connection with newly emerging drugs, screenshots of Daath’s distinctive blue website have frequently appeared in TV reports. It can be stated that Daath has become a recognised source of often exclusive drug information directly from the users.

### Challenges and outlooks

#### Focus challenges and outlooks

Pondering its future directions, Daath is clearly facing a dilemma about focussing its activities. One possible path is to return to the origins by pruning non-psychedelic topics (e.g., party drugs, legal highs, and designer substances). In this case, the website would probably lose a large number of – assumably younger – visitors and discussion participants who are currently drawn in because of their interest in new party drug use trends. However, a refocus on psychedelic drugs, entheogenic spirituality, and the psychonaut subculture would possibly increase discussion quality about these more abstract topics among the remaining participants.

The other direction would be to extend drug information and harm reduction by including all possibly used and misused substances, also non-psychedelic drugs (e.g., tobacco, alcohol, prescription medicines, stimulants, opiates, etc.). This comprehensive all-drugs approach has its pros and cons as well. Clearly, there would be a need for more harm-reducing drug information, as currently there is a lack of peer-help groups for most of the above listed non-psychedelic drugs—even though their lifetime prevalences and problem use rates are much higher as compared with psychedelic drugs. However, an extended website and discussion board may bring together previously isolated drug subcultures, thus it would actively and unintentionally draw attention to additional substances, including those associated with more problematic use. For novice drug users, it would be likely difficult to differentiate between risk levels of various substances, if high-risk drug use habits – such as intravenous injection – would be discussed as “normal” daily routines by some of the newly joining non-psychedelic members.

#### Social challenges and outlooks

Public attitudes in the Hungarian society are still overwhelmingly against drug decriminalisation and legalisation tendencies. This can be observed from public polls and media coverage as well as from angry anti-demonstrations and verbally violent online commentaries targeted at drug users. The fear of stigmatisation and social discrimination also hinders Daath’s efforts to register itself as a formal organisation, similar to drug users’ unions in the Netherlands and the Nordic countries. A viable possibility would be to organise the Daath community into a NGO not as a drug user advocacy group, but as one that provides merely drug information and harm reduction services. This alternative is currently under consideration by the most active members in the Daath community.

#### Commercial challenges and outlooks

Consistent to its low-profile policy, Daath has never been involved in any advertising activities, and it never promotes its name and/or logo to be displayed publicly. Due to its popularity and public image, the Daath brand may have a potentially high commercial value that is fully untapped at the moment. Thus, commercial pressures may arise occasionally upon realising how easily funds could be raised online from advertising revenues. Headshops, growshops, and smartshops would presumably be more than willing to display their product advertisements on the webpages of Daath, for those more than 1200 daily readers who are specifically interested in mind-altering substances. But as the costs of website operation are very low, there is no pressure on Daath to go commercial. In addition, Daath’s historical and current principles, and its persistent commitment to non-commerciality make no space for considering other options at the moment.

#### Legislative challenges and outlooks

Legislative challenges may arise with the long-expected reform of the criminal law, a change that is unpredictable in its content due to the currently turbulent political situation in Hungary. The currently repealed national drug strategy included and recommended also prevention and harm reduction measures, as well as it explicitly mentioned the involvement of drug user groups as relevant civil partners. However, it can be also pessimistically anticipated that an upcoming change in drug laws may restore the pre-2003 legal situation that criminalised the provision of certain drug-related information. For Daath, there would be a considerably higher risk to operate harm reduction services in such a hostile legal environment, where providing information for drug users might be considered punishable as “promoting drug use”. Still, recent changes in drug policies and attitudes at the international level may give a good reason for optimism for the Daath community to proceed and extend its harm reduction services and activities in the future.

## Conclusions

As an underground group of peer-helping drug users, Daath has to utilise a broad range of innovative measures in tackling with the difficult task of providing harm reduction without being accused of promoting drug use. Strongly embedded in the criminal and medical discourses, the operating environment seems to be more shaped by combating political ideologies, media-mongered moral panic, and covert economic interests than by evidence-based scientific knowledge and proven best-practice policies. On deeper moral-ethical levels, there are many unquestioned and unexplained substance-related taboos that society is reluctant to deal with. These taboos may result in reluctance to comprehensively reclassify all psychoactives substances of potential misuse by assessing their actual public health risks [28,29]. Another untouchable issue is the at least triple disproportion maintained by the public media: (1) overrepresenting atypically problematic cases of illicit drug use, (2) withholding discussion on serious problem use of legal “majority drugs”, and (3) suppressing discourses on possible use benefits of currently illicit drugs.

These seemingly consensual practices uphold the status quo paradigm, in which the use of scheduled substances is legally defined as “abuse”, i.e., a harm-producing activity that hence justifies harm-reducing measures to attenuate its negative consequences. While this may hold true in many cases, a realistic and comprehensive approach toward drug use should also include the vast majority of cases, where moderate drug use causes little to no problems over a longer time period (unless extended drug use itself is counted as a problem by definition). These cases call for a detailed examination of drug use purposes, which may also include another sensitive topic: the alleged benefits of drug use. Based on field observations, this topic is as self-evident for non-problem drug users as are drug-related risks: For many, drug use also includes a benefit-component as a motivating drive. It must be openly admitted that these two are often mixed within peer-help drug user groups: Hints and tips concern how to achieve both smaller minuses and bigger pluses. Therefore, any attempt to optimise this harm/benefit ratio should not only aim to decrease the harms of drug use, but simultaneously also to increase its benefits.

Hence, in parallel with harm reduction, a comprehensive model of drug use outcome modulation should also accept the – sometimes hardly distinguishable – concept of “benefit maximisation” [30]. The big question is, whether future directions in – a possibly reconceptualised, or even renamed – harm reduction are ready to accept and embrace this double-goal concept? It is yet to be seen in the future, whether current policies can remain enforced by the existing power structures, or will there be a paradigm shift toward a greater social acceptance of illicit drug use—starting with its least problematic forms. For the meanwhile, autonomous drug user groups – such as the Daath community – may still remain a source for innovative measures and tentative harm reduction best-practices to be assessed and possibly adapted also elsewhere.

## Abbreviations

2C-B: 2,5-dimethoxy-4-bromophenethylamine; 5-MeO-DMT: 5-methoxy-dimethyltriptamine; BZP: Benzylpiperazine; DMT: N,N-dimethyltryptamine; DXM: Dextromethorphan; EMCDDA: European Monitoring Centre for Drugs and Drug Addiction; GHB: γ-hydroxybutyric acid; GO: Governmental organisation; HCLU: Hungarian Civil Liberties Union; LSA: d-lysergic acid amide; LSD: Lysergic acid diethylamide; mCPP: Meta-chlorophenylpiperazine; MDMA: 3,4-methylenedioxy-methamphetamine; N2O: Nitrous oxide; NGO: Nongovernmental organisation; NSD: New synthetic drugs; THC: Δ-9-tetrahydrocannabinol

## Competing interests

The authors declare that they have no competing interests.

## Authors’ contributions

LM observed the case group while participated in its activities, and drafted the manuscript. JR provided institutional and professional support, and revised the manuscript. Both authors read and approved the final manuscript.

## Authors’ information

LM is a PhD student of the Consciousness Research Group at the Centre for Cognitive Neuroscience, University of Turku, Finland. His main research interests focus on altered states of consciousness, such as dreaming, hypnosis, hallucinations, and psychoactive drugs.

JR is a professor at the Department of Addiction Medicine, Faculty of Health Sciences, Semmelweis University, Budapest, Hungary. He is also a professor at the Institute for Psychology of the Eotvos University, Budapest, Hungary. By clinical practice he is a psychiatrist and director of the Blue Point Drug Counseling and Outpatient Center.
